# Diabetic Gastroparesis and its Emerging Therapeutic Options: A Narrative Review of the Literature

**DOI:** 10.7759/cureus.44870

**Published:** 2023-09-07

**Authors:** Shiza A Zahid, Ritu Tated, Midhun Mathew, Daniel Rajkumar, Siddhant B Karnik, Akshara Pramod Roy, Fredy P Jacob, Rishabh Baskara Salian, Waleed Razzaq, Divya Shivakumar, Uzzam Ahmed Khawaja

**Affiliations:** 1 Department of Internal Medicine, Jinnah Sindh Medical University, Karachi, PAK; 2 Department of Internal Medicine, Mahatma Gandhi Mission Institute of Medical Sciences, Navi Mumbai, IND; 3 Department of Internal Medicine, Pennsylvania Hospital, Philadelphia, USA; 4 Department of Internal Medicine, Hospital Alor Gajah, Alor Gajah, MYS; 5 Department of Internal Medicine, Lokmanya Tilak Municipal Medical College and General Hospital, Mumbai, IND; 6 Department of Family Medicine, Roy's Heart Foundation, Chennai, IND; 7 Department of Internal Medicine, Jonelta Foundation School of Medicine, University of Perpetual Help System DALTA, Las Piñas, PHL; 8 Department of Internal Medicine, Kasturba Medical College of Manipal, Mangalore, IND; 9 Department of Internal Medicine, Services Hospital Lahore, Lahore, PAK; 10 Department of Internal Medicine, Kamineni Academy of Medical Sciences and Research Center, Hyderabad, IND; 11 Department of Paediatrics and Child Health, Aga Khan University Hospital, Karachi, PAK

**Keywords:** psychological intervention, quality of life, glycemic control, relamorelin, tradipitant, diabetic gastroparesis

## Abstract

Diabetic gastroparesis (DG) is one of the many complications of diabetes mellitus (DM). Even though this condition surfaces years after uncontrolled disease, it affects the quality of life in several ways and causes significant morbidity. Common symptoms experienced by the patients include postprandial nausea, vomiting, abdominal fullness, and pain. Strict glycemic control is essential to evade the effects of DG. The purpose of this review article is to briefly study the pathophysiology, clinical features, diagnostic modalities, and the effects of DG on different aspects of life. Furthermore, it also focuses on the emerging treatment modalities for DG. Tradipitant and relamorelin are two such treatment options that are gaining noteworthy recognition and are discussed in detail in this review article. As observed through various clinical trials, these drugs help alleviate symptoms like nausea, vomiting, abdominal pain, and bloating in patients suffering from DG, thereby targeting the most common and bothersome symptoms of the disease. This leads to an improvement in the quality of life, making it a reliable treatment option for this disease. But while pharmacological intervention is vital, psychological support and lifestyle changes are equally important and are the reason why a multidisciplinary approach is required for the treatment of DG.

## Introduction and background

Diabetic gastroparesis (DG) is a complication of diabetes that results in delayed emptying of gastric contents and is associated with upper gastrointestinal symptoms without any gastric outlet obstruction [1]. The common symptoms associated with DG are delayed emptying, abdominal pain, anorexia, weight loss, postprandial fullness, and vomiting [2]. Among diabetic patients, approximately 75% have associated gastrointestinal symptoms [3], and about 18% experience upper gastrointestinal symptoms [4]. In order to meet the diagnostic criteria for DG, gastrointestinal symptoms should persist for over three months [5]. DG is usually seen in patients with poorly controlled diabetes (blood glucose >200 mg/dL), due to dysregulation in the coordination between the autonomic nerves, the pacemaker cells, and the intestinal musculature [6]. Delayed gastric emptying can cause a substantial reduction in the quality of life (QoL) as it results in poor glycaemic control, which is associated with complications, abdominal discomfort, and poor nutrition, resulting in increased hospitalizations and ultimately leading to psychological distress in the individual [2]. According to the latest International Diabetes Federation (IDF) report, an estimated 537 million adults worldwide between the ages of 20-79 years are diagnosed to have diabetes; this number is expected to reach 643 million before 2030 [7]. Research on the epidemiology of gastroparesis was conducted in Minnesota between 1996 to 2000. The incidence per 100,000 was 9.8 in females and 2.5 in males. The prevalence per 100,000 was 37.8 in females and 9.6 in males [8]. A population-based survey conducted in the U.S. in 2020 revealed that the prevalence of gastroparesis is 1.5 times higher in males compared to the female population across all ages [9]. Although most of the etiologies of gastroparesis are idiopathic, diabetes is the single most common identifiable disease that is significantly related to this condition. About one-third of patients with diabetes develop DG [2,10]. According to the Gastroparesis Clinical Research Consortium (GpCRC), an estimated 5 million people in the US suffer from DG [11]. A cohort study conducted by Kofod-Andersen revealed the prevalence of DG in type 1 diabetic individuals to be 9.8% [12].

According to a cohort study in the United States, the incidence of patients suffering from gastroparesis over the last decade is identified to be 5.2 % and 1 % in type 1 and 2 diabetes respectively [13]. Patients suffering from type 1 diabetes are 3.5 times more likely at risk of having gastroparesis than type 2 diabetes. However, the cumulative figure of patients presenting with gastroparesis is comparatively higher in type 2 diabetes due to the vast prevalence [9,14].

## Review

Normal physiology of the gastrointestinal system

The neurophysiology of the gastrointestinal tract is divided into an intrinsic (enteric) nervous system and an extrinsic nervous system (CNS). The enteric nervous system is a subdivision of the autonomic nervous system that is specifically equipped with intrinsically integrated circuits that allow the synchronization of gastrointestinal functions with no feedback from the CNS. It’s formed by an integrated network of neurons and enteric glial cells (EGC) in the wall of the entire tract [15]. The intrinsic primary afferent neurons (IPANs) are specialized sensory cells that function to modulate the working and balance of the GI system but hyperglycemia has a major effect on the EGCs and enteric neurons [15]. The CNS regulates enteric behavior, and the stomach also transmits information to the brain, thus while the ENS is capable of functioning independently from the CNS, it typically does not. Most of the vagal fibers connecting the stomach and brain are afferent, indicating that the brain is primarily a receiver when it comes to communication with the gut as opposed to a transmitter [16]. 

The CNS directly innervates the esophageal striated muscle leading to both mastication and swallowing being CNS-dependent. Extrinsic neural control of upper gastrointestinal motor function is provided by the vagal parasympathetic outflow, which is excitatory to non-sphincteric muscle, as well as the thoracolumbar sympathetic supply, which is excitatory to sphincters and inhibitory to non-sphincteric muscle. The enteric nervous system contains a larger myenteric Auerbach’s plexus located in the muscularis externa and a secretory Meissner’s plexus located in the submucosa. The intestinal lumen or its epithelial lining is not really accessed by nerve fibers, but the mesentery and the blood vessels allow the extrinsic innervation of the nerve [16]. Digestion is coordinated in a phasic manner with the integration of numerous signals from the ENS and CNS; neural signals also pass between distinct gut regions to synchronize digestive activity [16]. Interstitial cells of Cajal (ICCs) take part in various functions of the gastrointestinal tract. They are non-neuronal cells that act as a pacemaker, generating electrical activity that causes the gut to move in slow waves and are involved in neurotransmission, determining the smooth muscle membrane potential gradient, and the mechanical transmission of the impulse [17]. Recent research has begun to elucidate the intricate processes that govern ICC networks in the gut, as well as their dysfunction in gastroparesis. ICC networks are normally constantly rebuilt and maintained by a balance of mechanisms that harm and preserve these cells [17].

**Figure 1:**
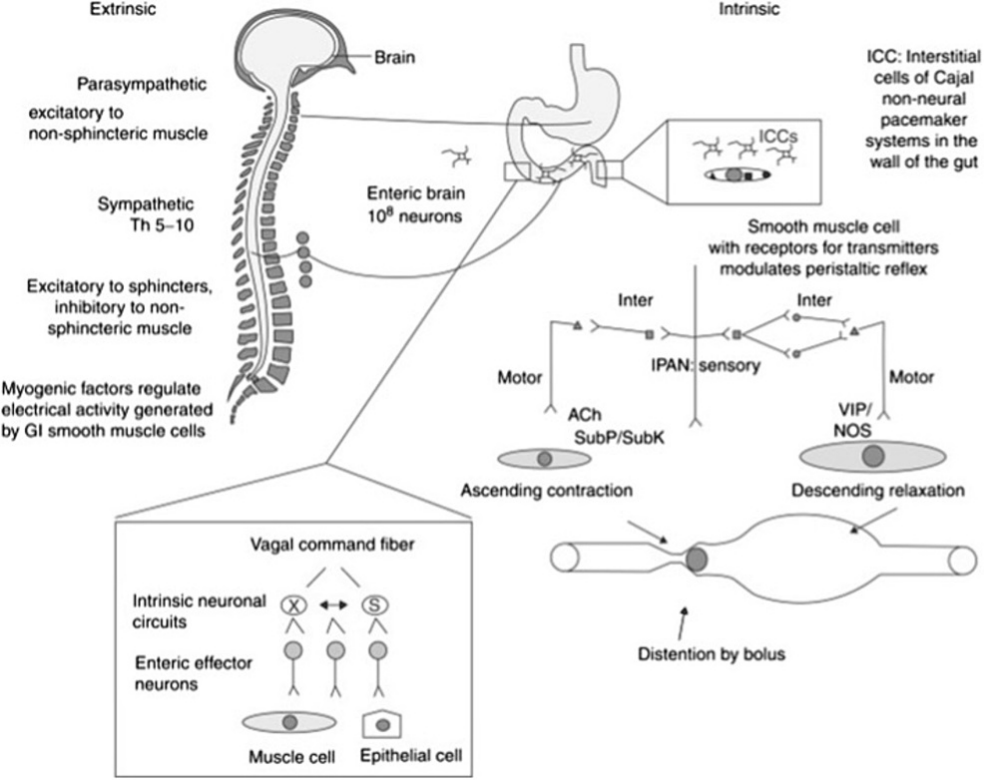
Schematic Demonstration of the Extrinsic (CNS) and Intrinsic (Enteric) nervous system. Reproduced with permission from Quigley [21]. Copyright 2017 Elsevier.

They also engage in the transmission of signals across enteric motor neurons, efferent from the central nervous system, and smooth muscle cells in the GI tract wall [18]. Fundic tone and antral contractions are coupled with suppression of pyloric and duodenal contractility whilst gastric emptying, as well as an association between smooth muscle, enteric and extrinsic autonomic neurons; the Cajal interstitial cells are required for gastric emptying [19]. Neurotransmitters involved in the propagation of impulses are acetylcholine, substance P, and neurokinin, which are released from the enteric neurons and attach to the receptors present on the ICCs in the muscular layer of the tract [20]. However, it is crucial to note that the whole process of neurotransmission doesn’t exclusively depend on the ICCs. Nitric oxide (NO) is exclusively involved in inhibitory neurotransmission [20]. Figure 1 below shows an overview of the gastrointestinal tract being controlled by the nervous system.

**Figure 2:**
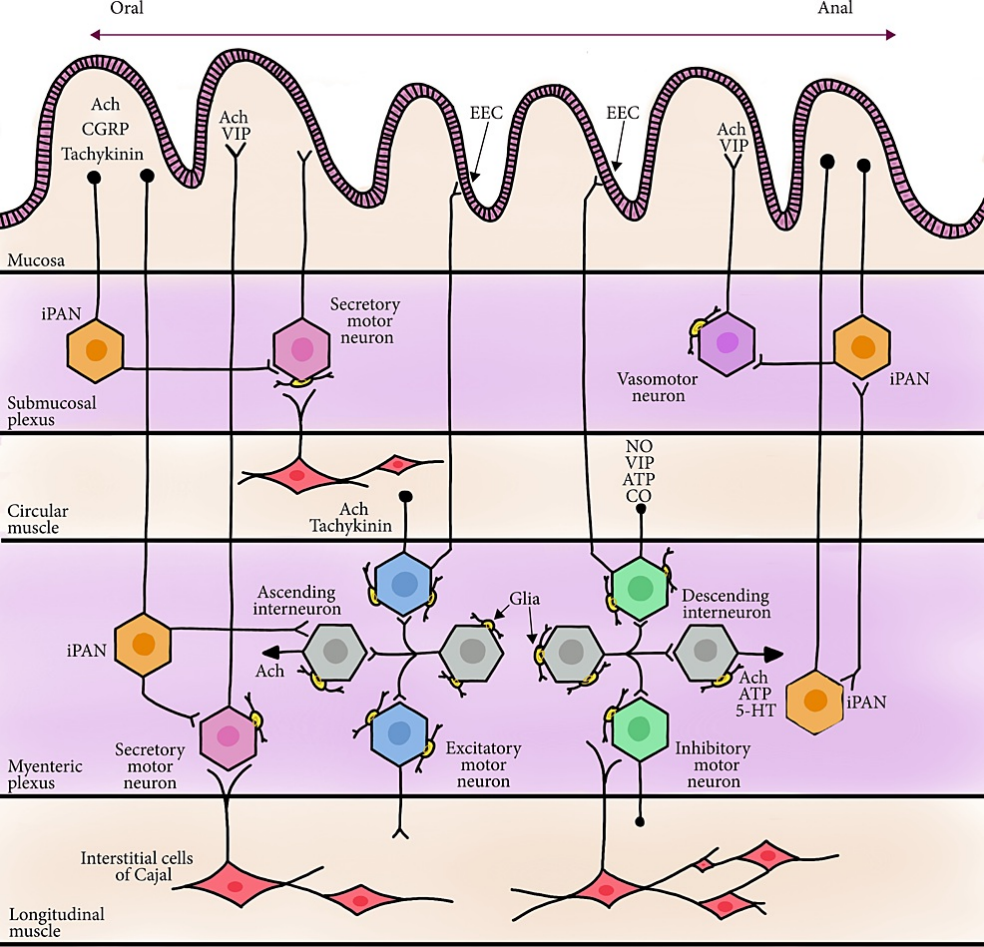
Connections involved in local enteric reflexes illustrated graphically. Reproduced under the Creative Commons Attribution License from ref [22]. Copyright 2020 Mark A. Fleming II et al.

Figure 2 attached below illustrates the internuclear connections of cells in the gastrointestinal tract.

Intestinal changes in diabetic gastroparesis

Gastroparesis is a condition characterized by slowed gastric emptying (GE) and symptoms that are not related to gastric obstruction. The GI symptoms are contributed by the small intestine in addition to the stomach. Diabetic gastroenteropathy is multifactorial and involves not only parasympathetic and sympathetic autonomic nerves, but also enteric neurons, smooth muscle cells, and ICCs [19]. In addition to the factors mentioned above, delayed gastroenteropathy in diabetes mellitus (DM) is associated with hyperglycemia; incretin-based medications are used to stabilize postprandial blood glucose. Additionally, psychological factors may be responsible for diabetes-related gastric motor dysfunction [23]. Research studies performed on humans demonstrate noteworthy loss of ICCs in the antrum of one-third of patients in refractory gastroparesis [24]. Insulin growth factor-1 and insulin deficiency have also been implicated in the loss of ICCs. It is well understood that diabetes is a state of extreme oxidative stress and when the mechanisms counteracting the stress are weakened, it leads to the death of ICCs [25]. One such example is the presence of heme oxygenase-1 in the macrophages, which increases carbon monoxide [26] in response to stress, leading towards an indirect rise in nNOS (neuronal nitric oxide synthase) and ICCs, which regularize delayed gastric emptying [24]. The environment within the enteric nervous system is considerably affected by inflammation. One such study on sham feeding has demonstrated decreased pancreatic polypeptide and gastric secretions in response to vagus nerve stimulation in diabetes [27].

The mechanisms through which diabetes affects the enteric neurons are well explained by the research conducted on animal models with streptozotocin-induced DM [28]. The vagus nerve, which conducts autonomic afferent and efferent signaling, forms extensive communication with the brain. There has been concrete evidence of parasympathetic and sympathetic ganglia of the vagus nerve being affected, leading to anatomical variations and contributing to decreased GI function [29]. As for the structural changes, the vagal nerve fibers demonstrate signs of “segmental demyelination” and “axonal degeneration” in the myenteric and submucosal plexus [30]. Diabetes especially affects inhibitory neurons more as compared to excitatory neurons. Nitrergic neurons are affected quickly following the onset of DM in animal models and nNOS-expressing cells are decreased in diabetic animals while there is no change observed in the number of cholinergic neurons until later in diabetes [30]. Another study conducted on humans with diabetes demonstrated fewer neurons with an inhibitory neurotransmitter, Neuropeptide Y [31]. 

The way in which diabetes affects the neurons in the gastric tract is not well-defined even now. There are several mechanisms that have been proven to contribute to GI symptoms. Studies conducted on animal models have proven hyperglycemia to be a crucial factor in the apoptosis of enteric cells [32]. Neurons migrate from the sacral and vagal neural crest and express RET tyrosine kinase receptor, an important substance for the differentiation and growth of enteric cells. A growth factor, glial cell line-derived neurotrophic factor (GDNF) is known to stimulate the RET receptor by means of PI3K and MAPK pathways [33].

Excessive amount of dietary fat intake has been consistently associated with the loss of myenteric neurons, palmitic acid contributing to the shrinking of neurons, chromatin concentration, and a major reduction in neuronal survival [34]. BMAP/SMAD signaling pathway has also been linked as one of the mechanisms as abnormal bone morphogenic proteins were found in diabetic rat models [35]. Extra reactive oxygen species (ROS) causing oxidative stress have also been seen as a likely cause of enteric neuron degeneration due to DM. In animal models of DM, high levels of oxidative stress have been disclosed to be related to gastroparesis, and the use of antioxidants is able to inhibit the progress of gastroparesis [25]. A recent study showed evidence of reduced levels of the antioxidant glutathione and comparatively high quantities of superoxide dismutase mRNA, signifying stress [28]. Another article implied that myocytes affected in GI tracts of streptozotocin-induced diabetic rats have shown high expression of Na-K+-ATPase pump with elevated calcium levels intracellularly [36]. Another research article using streptozotocin-induced diabetic animal models demonstrated that dysfunction in GTP-binding protein may have an impact on the delayed motility of the GI tract [37]. 

Additional studies have shown that diminished expression of myosin light chain kinase is correlated to GI motility in streptozotocin-induced rats, which can be undone by the administration of insulin [38]. The depletion of bacteria in the gut causes significant distension of the caecum owing to reduced motility [39]. In some studies, it has been perhaps established that bacteria are crucial for the formation of short-chain fatty acids, which when decreased lead to dysmotility. The role of microRNAs, which are posttranslational protein regulators, have also been found to play a role in the growth of smooth muscle cells in the GI tract, but not much evidence is available to link them to diabetic changes in the gut [40]. Moreover, few studies have also shown how females are highly dependent on nitrergic neurons for gastric motility, and hence loss of nNOS affects female rats more than male rats [41]. Autoimmune infiltrates have been found in specimens of the esophagus [42], but gastric biopsies haven’t shown any concrete evidence [43].

Impact of clinical features of diabetic gastroparesis on quality of life

DG is a chronic condition of gastric motility that causes the stomach to take longer than usual to empty either solids or liquids when there is no physical obstruction. Although it has a low incidence rate, DG often occurs in patients with long-standing diabetes and lowers the quality of life (QoL) while raising mortality and hospitalization rates [44]. DG symptoms might be modest or incapacitating. Early satiety, postprandial fullness, bloating, nausea, and a significantly larger abdomen are all symptoms of gastroparesis [44]. For many people, pain is significant but is likely underreported; 72% of gastroparesis patients reported having stomach pain [45].

Patients with DG had poorer SF-36 physical and mental component scores, which indicates a lower QoL [46]. Poor QoL is reported in gastroparesis patients as suggested by several measurements. QoL is impacted by a number of factors, including symptoms like nausea, vomiting, and stomach pain, comorbidities such as psychological issues like anxiety and depression, and patient-related issues such as smoking. In cases of gastroparesis, focusing on the modifiable factors may improve patient outcomes [47]. Overall, gastroparesis has a similar effect on QoL as active inflammatory bowel disease [48,49]. The mental aspect of the QoL in DG is lower than that in rheumatoid arthritis patients, but the physical QoL is comparable [50].

Mortality and hospitalization rates for diabetic gastroparesis

In a case series, the estimated rate of mortality for gastroparesis ranged from 38% at four years to 34% at 25 years [51-56]. The survival rate in patients with gastroparesis was significantly lower in the overall population compared to tertiary centers or communities [8,56]. The five-year survival adjusted for age and sex was estimated to be 67% (95% CI) in gastroparesis compared to 81% in the community population. Those with idiopathic gastroparesis have better survival rates than those with non-idiopathic gastroparesis, wherein non-idiopathic is mostly comprised of diabetic gastroparesis [8]. The survival of those with postinfectious and idiopathic gastroparesis in a tertiary referral is better than those with gastroparesis from diabetes and post-surgery [56]. 

Gastroparesis can be considered as a marker for increased morbidity. Cardiovascular disease, hypertension, and retinopathy are more likely to be present in patients with type 1 and 2 DM presenting with symptoms of gastroparesis like nausea, vomiting, early satiety, retching, bloating, and a documented delay in gastric emptying [57]. On the contrary, in a cohort, when corrected for HbA1c and autonomic neuropathy, diabetic gastroparesis had a good prognosis and was not associated with an increase in mortality in patients with type 1 DM when followed over a period of 25 years [53]. In patients with diabetic gastroparesis, an increase in mortality can be explained by associated comorbidities due to DM [58]. In patients with gastroparesis, the inpatient mortality was 0.25%. Patients with non-diabetic gastroparesis had higher odds of inpatient mortality compared to patients hospitalized due to gastroparesis. They also had a longer duration of hospital stay, higher mean total healthcare cost, and higher odds of system-based complications like PE, DVT, and sepsis [59]. 

The rate of hospitalization due to diabetic gastroparesis has increased over the years. According to a study conducted between 1995 and 2004, hospitalization due to gastroparesis has risen by 138%, and hospitalization rate due to gastroparesis as a primary diagnosis increased by 158%. Among these patients, the proportion of those who had DM increased from 21% in 1995 to 26.7% in 2004. The changes in DM-related hospitalizations (+53%), all hospitalizations (+13%), and hospitalizations with four other GI conditions, i.e., gastroesophageal reflux disease (GERD), gastric ulcer, gastritis, or nonspecific nausea/vomiting as the primary diagnosis (−3% to +76%) was lesser than the corresponding hospitalizations due to gastroparesis [60]. Several factors influence the rise in the incidence of hospitalization due to gastroparesis. These include raises in the prevalence of DM, severity, changes in criteria for diagnosis, treatment approach, better recognition/diagnosis of the disorder, and changes in hospital coding practices [58]. In the National Institute of Diabetes and Digestive and Kidney Diseases (NIDDK) consortium study, hospitalization for gastroparesis due to type 1 DM were more (i.e., 5.1 ± 6.4 per year (mean ± SD)), mostly from dehydration and vomiting, than for those with idiopathic (1.6 ± 3.0) or due to type 2 DM (2.7 ± 5.7) (Table 1) [61]. In another study, the duration of hospitalization among the patients with DM was much higher in those who had delayed gastric emptying compared to those with normal gastric emptying (i.e., 25.5 vs. 5.1 per 1000 patient-days) [57]. In patients with type 1 DM, the HbA1c levels, duration of symptoms, rate of hospitalizations, and gastric retention were much higher than type 2 DM. Gastroparesis patients using opioids may develop worsening symptoms, increased gastric retention, poor healthcare utilization, and poor QoL [62]. A study of 48-week follow-up in those with type 1 DM had an increase in usage of prokinetic, PPI, anxiolytic and also an increase in usage of gastric stimulator implantation rate [63]. Symptoms that are refractory can lead to recurrent hospitalization. In a study, it was found that the rate of readmissions for gastroparesis was substantial (26.8% at 30 days and 45.6% at 90 days). There was a higher risk of readmissions in patients with comorbidities (DM) and long initial hospitalization [64].

**Table 1:** Treatment, hospitalization, comorbidities, psychological function, and quality of life. *Values are mean ± standard deviation or n (%). Id=Idiopathic, DM=Diabetic, T1DM=Type 1 Diabetic, T2DM=Type 2 Diabetic †P values (two-sided) determined from either a chi-square test for non-ordered categories, a Fisher’s exact test or the Cochran-Armitage trend test for ordered categories for categorical variables, or a Kruskal-Wallis test for continuous variables. ‡Any pain relieving medication includes any analgesics, non-steroidal anti-inflammatory (NSAI), or aspirin medications taken in the past six months. §Subscales derived from the Patient Assessment of Upper Gastrointestinal Disorders-Quality of Life (PAGI-QOL). Scales have been recoded so that a higher score reflects a higher QOL. Scores on the Medical Outcomes Study 36-Item Short-Form Health Survey V2 (SF-36v2) standard recall were normalized to the 1998 U.S. general population with a mean (± SD) of 50± 10, except for the Health Transition item. A higher score reflects higher QOL or better health outcome. Reproduced with permission from Parkman et al [61]. Copyright 2011 Elsevier.

	Idiopathic	Type 1 Diabetic	Type 2 Diabetic	Pairwise P value†		
	(N=254)	(N=78)	(N=59)	Id vs.	Id vs.	Id vs.
Characteristics	N (% or mean)*	N (% or mean)*	N (% or mean)*	All DM	T1DM	T2DM
Medications use (current) and treatment						
Proton pump inhibitors, other GI meds	193 (76.0%)	62 (79.5%)	49 (83.1%)	0.25	0.52	0.24
Prokinetic meds for gastroparesis	125 (49.2%)	54 (69.2%)	38 (64.4%)	<0.001	0.002	0.04
Antiemetics for gastroparesis	154 (60.6%)	55 (70.5%)	39 (66.1%)	0.12	0.11	0.44
Any NSAI pain relieving in past 6 months‡	153 (60.2%)	42 (53.9%)	40 (67.8%)	0.94	0.32	0.28
Narcotic pain med	109 (42.9%)	36 (46.2%)	28 (47.5%)	0.47	0.61	0.53
Any pain modulators	42 (16.5%)	21 (26.9%)	17 (28.8%)	0.009	0.05	0.03
Any antidepressants	83 (32.7%)	30 (38.5%)	20 (33.9%)	0.45	0.35	0.86
Any anxiolytics	39 (15.4%)	7 (9.0%)	11 (18.6%)	0.55	0.15	0.53
Any estrogen, progestin, HRT	60 (23.6%)	12 (15.4%)	12 (20.3%)	0.16	0.12	0.59
Has gastric electrical stimulator (yes vs no)	15 (5.9%)	12 (15.4%)	2(3.4%)	0.12	0.01	0.75
Hospitalizations & comorbidities:						
Any hospitalization in past year	112 (44.1%)	57 (73.1%)	27 (45.8%)	<0.001	<0.001	0.82
Average number of hospitalizations past year	1.6 ± 3.0	5.1 ± 6.4	2.7 ± 5.7	< 0.001	< 0.001	0.59
Average number of comorbidities	3.7 ± 2.8	4.0 ± 2.8	5.6 ± 3.5	0.003	0.40	< 0.001
Comorbidity, ever diagnosed (yes vs no)						
Endometriosis	40 (15.6%)	4 (5.1%)	7 (11.9%)	0.03	0.01	0.45
Cholelithiasis or any gallbladder disease	89 (35.0%)	21 (26.9%)	25 (42.4%)	0.77	0.18	0.29
Migraine headaches	103 (40.6%)	19 (24.4%)	22 (37.3%)	0.04	0.01	0.64
Major depression	54 (21.3%)	22 (28.2%)	19 (32.2%)	0.06	0.20	0.07
Severe anxiety disorder	31 (12.2%)	8 (10.3%)	5(8.5%)	0.42	0.64	0.42
Beck Depression Index (BDI) (past 2 weeks)						
Inventory score	18.7 ± 11.0	21.6 ± 12.9	18.6 ±1 0.0	0.78	0.11	0.76
Score>28 (severely depressed)	47 (18.5%)	20 (25.6%)	10 (17.0%)	0.42	0.17	0.78
Feelings of hopelessness	26 (10.2%)	15 (19.2%)	8 (13.6%)	0.06	0.03	0.46
State-Trait Anxiety Inventory (STAI)						
State anxiety score	45.2±13.4	47.7±14.1	44.6±12.9	0.37	0.13	0.77
State anxiety score ≥ 50 (severe)	91 (35.8%)	37 (47.4%)	18 (30.5%)	0.40	0.07	0.44
Trait anxiety score	43.9±12.1	47.3±12.7	43.7±12.9	0.16	0.03	0.83
Trait anxiety score ≥ 50 (severe)	87 (34.3%)	35 (44.9%)	17 (28.8%)	0.47	0.09	0.42
Quality of Life (PAGI-QOL) (past 2 weeks) §						
Daily activities sub-score	2.2 ± 1.2	2.2 ± 1.3	2.4 ± 1.2	0.99	0.46	0.37
Clothing sub-score	2.9 ± 1.8	3.0 ± 1.7	3.0 ± 1.7	0.67	0.73	0.75
Diet sub-score	1.4 ± 1.2	1.7 ± 1.2	1.8 ± 1.3	0.002	0.01	0.01
Relationship sub-score	2.9 ± 1.5	2.8 ± 1.7	3.1 ± 1.5	0.91	0.53	0.35
Psychological sub-score	2.7 ± 1.4	2.4 ± 1.5	2.8 ± 1.4	0.36	0.07	0.58
Total score	2.4 ± 1.1	2.4 ± 1.1	2.6 ± 1.2	0.50	0.88	0.19
SF-36v2 Health Survey (past 4 weeks) §						
Current general health perception	32.3 ± 9.8	26.6 ± 7.4	27.9 ± 6.7	<0.001	<0.001	0.002
Emotional problems limit your daily work	36.9 ± 15.2	32.1 ± 14.8	33.0 ± 15.7	0.006	0.01	0.08
Physical health component summary score	34.0 ± 10.3	32.6 ± 10.0	29.6 ± 9.2	0.02	0.31	0.005
Mental health component summary score	37.6 ± 12.5	34.2 ± 12.4	37.3 ± 13.0	0.10	0.03	0.85

Diagnostic modalities for diabetic gastroparesis

The diagnosis of gastroparesis is based upon both recognition of the cardinal symptoms of gastroparesis and identification of delayed gastric emptying by appropriate instruments. Numerous techniques are available for studying gastric emptying but the gold standard test for diagnosing gastroparesis is gastric emptying scintigraphy (GES) [65]. The upper gastrointestinal tract symptoms suggestive of gastroparesis should be extensively investigated as other disorders result in similar symptoms including peptic ulcer disease, gastric cancer, pancreatic or biliary disease, gastroesophageal reflux disease (GERD), and gastric outlet or mechanical small bowel obstruction. Diabetic gastroparesis also needs to be differentiated from chronic gastric stasis as a result of previous surgery, metabolic and endocrine disease (liver or renal disease and thyroid dysfunction), CNS disorders (brain tumor, stroke or trauma), malignancy, and HIV infection [66]. It is also important to discontinue all drugs that can delay gastric motility (e.g. opioids and anticholinergics) or accelerate gastric motility (e.g. prokinetics and erythromycin) at least 48-72 hours before the study. The blood sugar should also be closely monitored as hypoglycemia and hyperglycemia are associated with accelerated and delayed emptying, respectively and the current guidelines recommend not to do the testing if blood sugars are >275 mg/dl [67]. In the following sections, we discuss various modalities of investigation for diabetic gastroparesis such as wireless motility capsule (WMC) and breath testing that can objectively demonstrate delayed gastric emptying [68].

Gastric Emptying Scintigraphy (GES)

GES is the gold standard test for reliable assessment of gastric emptying. The Society of Nuclear Medicine and Molecular Imaging (SNMMI) and the American Neurogastroeneterology and Motility Science (ANMS) recommend a four-hour test using 99mTechnetium-radiolabeled egg white meal with jam, toast, and water for testing with imaging with a gamma camera at zero, one, two and four hours after meal ingestion. For this meal delayed gastric emptying is defined as >60% retention in two hours and/or 10% at four hours. Moderate to severe GE is defined as >20% retention at four hours [58]. The limitation of GES is that patients are exposed to a modest dose of radiation, and the test is relatively expensive and confined to specialized centers [66].

Gastric Emptying Breath Test (GEBT)

Breath test is an alternative to scintigraphy where we measure nonradioactive 13CO2 in exhaled breath samples after ingestion of a 13CO2 labeled meal. The non-radioactive isotope carbon protein is used to label octanoate and medium-chain triglyceride which can be bound into a solid meal. After ingestion and emptying from the stomach, octanoate is absorbed by the small intestine and is metabolized to carbon dioxide in the liver. The radiolabelled 13C is excreted from the lungs during expiration. A mass spectrometer is used to measure the 13C labeled CO2 in end-tidal breath samples. The rate-limiting step of the exclusion of the 13C is gastric emptying. Breath samples are obtained periodically over several hours. The exhaled 13CO2 represents the rate of gastric emptying, duodenal absorption, hepatic metabolism, and pulmonary excretion [69]. GBT has a sensitivity of 89% and specificity of 80% for identifying delayed gastric emptying. Gastric emptying using the breath testing results correlates well with the results of the gastric emptying scintigraphy [70].

Wireless Motility Capsule (WMC)

WMC (SmartPill Wireless Motility Capsule, SmartPill Corp., Buffalo, NY)) is an FDA-approved device for the evaluation of gastric emptying in patients with gastroparesis. The device is a 2.6-millimeter ingestible capsule that transmits the temperature, pH, and pressure data to the wireless device worn by the patient. The capsule typically passes through the digestive tract in two to five days after which the data are downloaded. Gastric retention time is determined by measuring the change in pH as the WMC passes from the gastric antrum to the duodenum. WMC may offer an alternative to scintigraphy in patients wishing to avoid radiation as much as possible. WMC is contraindicated in patients with a non-gastrointestinal stricture [71]. It has a sensitivity of 59% to 86% and a specificity of 64% to 81% [72].

Ultrasonography

Gastric ultrasonography has been used to assess antral wall motion, patterns of transpyloric flow, and gastric emptying based on changes in the cross-sectional area or diameter of the gastric antrum. Gastric ultrasonography is noninvasive, safe, cheap, and widely available and allows for bedside monitoring and does not expose the patient to ionizing radiation which is restricted particularly in children and pregnant women, and shows reasonably good interobserver agreement in evaluation of liquid gastric emptying. However, ultrasonography is unable to distinguish between the solid and liquid components of a meal and therefore is unsuitable to assess the emptying of solids. Ultrasonography also requires an experienced technician and is user dependent and may be influenced by the presence of intragastric air or posture and is generally considered impractical for prolonged observations [72].

Electrogastrography

Electrogastrography, non-invasively measures gastric myoelectric activity by placing surface electrodes on the epigastric skin. The normal frequency of gastric slow wave is about three cycles per minute. Spectral analysis is used to identify the dominant frequency and electrical activity that is within, below (bradygastria), or above (tachygastria) the normal range. Although bradygastria and tachygastria are considered abnormal, they also occur in healthy people and the measurement of electrical activity is interfered with by electrical activity from other organs. This test is not yet standardized and is predominantly used as a research tool rather than for clinical purposes [58,66].

Factors affecting diabetic gastroparesis 

Relationship Between Glycaemic Control and Diabetic Gastroparesis 

Even in healthy individuals, hyperglycemia (blood glucose >200mg/dL) exerts its inhibitory effect on gastric emptying (GE) by reducing antral motility and pyloric pressure waves [73,74]. GE and glycaemic control in DM have a bidirectional relationship [75]. Hyperglycaemia leads to slower GE, whereas acute insulin-mediated hypoglycemia (blood glucose 2.0-2.6 mmol/l) tends to hasten the rate of GE in type 1 diabetics, even in those suffering from cardiovascular autonomic neuropathy and gastroparesis secondary to DM [76]. Similarly, the rate of GE affects glucose levels, which is most noticeable after a meal [77,78]. HbA1c levels, in an individual, reveal the extent of glycaemic control for the past three months. There is varying data on the relationship between HbA1c levels and GE. A higher HbA1c concentration is associated with a higher rate of gastrointestinal symptoms and delayed GE among patients with DM, as evidenced by a few cross-sectional studies. However, several other factors, in addition to an elevated HbA1c, account for this finding which includes the duration of DM and mean HbA1c for the past 27 years [79]. 

Strict maintenance of blood glucose levels within the normal range improves retinal, renal, and neural symptoms in type 1 DM and even in type 2 DM but to a lesser degree [80,81]. However, improved glycaemic control didn’t have much of an effect on GE in patients with type 2 and poorly controlled DM [82]. Nevertheless, in the majority of cases, it has been observed that only a modest delay in GE is seen in patients with DM who go on to develop gastroparesis [1,82]. While no change is seen in the rate of GE for the initial 25 years following the diagnosis of DM [83].

Impact of Nutrition and Hydration on Diabetic Gastroparesis

As concluded by various small clinical trials, it is best to have small portions and to have a low-fat and low-fiber diet for patients suffering from gastroparesis [84,85]. Instead of consuming a single fulfilling meal, small frequent meals are recommended to avoid post-prandial bothersome symptoms. In a survey performed on 12 patients suffering from gastroparesis (three with DG) demonstrated that symptoms were most prominent after a solid diet rich in fat, followed by diets with low-fat solids, high-fat liquids, and low-fat liquids [86]. Similarly, fatty, acidic, spicy, and roughage-rich foods produce more prominent symptoms as compared to sweet, salty, bland, and starchy foods [87]. Another approach for nutrition in patients with DG is to introduce foods in a stepwise manner starting from liquids to smoothies and soups, and ultimately transitioning to gastroparesis-compliant solid food [88]. If all interventions fail, the last resort is to consume calorie-rich liquids as digestion of liquids is mostly preserved in patients with DG [2]. 

Role of Depression in Diabetic Gastroparesis

Patients with gastroparesis frequently report psychological symptoms like depression, anxiety, and impaired QoL [89-98]. The prevalence of these symptoms is increased in patients with type 2 DM. In general, GI symptoms are influenced by psychological factors such as anxiety and depression but not much is known about this association in diabetics [99]. However, it has been noted that the use of antiemetic and prokinetic drugs in managing gastroparesis symptoms is associated with higher depressive scores [100]. The reported prevalence of these psychopathologies is combined anxiety/ depression at 24% [95], severe anxiety at 12.4% [93], depression at 21.8-23% [51,93], somatization at 50% [51], and others at 5% [95].

Psychological Interventions to Manage Diabetic Gastroparesis

Only one study has reported regarding a psychological intervention for gastroparesis patients. Liu et al. mentioned that both depression scores and gastric functions improve significantly in patients who undergo psychological interventions compared to patients who receive standard care, however, the study had considerable methodological limitations [101]. Firstly, the study also used several different interventions including supportive mental consultation, abdominal massage, music, etc. making it impossible to ascertain the impact of any one component of the intervention. Additionally, the study also did not exercise long-term follow-up [101].

Although the results of the study are promising, there is still limited evidence for the use of psychological intervention in gastroparesis and measures of other important psychological factors such as anxiety and QoL have yet to be assessed.

In conclusion, measures of depression and anxiety correlate with gastroparesis severity as determined by investigator-graded and patient-reported assessments. Psychological dysfunction does not vary by disease etiology or by the magnitude of gastric retention. Future longitudinal evaluations will provide a greater understanding of psychological dysfunction in gastroparesis and if directed, therapy to manage depression and anxiety can reduce gastrointestinal manifestations of this disorder. Nevertheless, the findings of this study suggest that both physical and psychological features should be considered in developing individualized treatment plans for gastroparesis [100]. 

Emerging therapeutics for diabetic gastroparesis

Tradipitant

Drug class: Tradipitant (VLY-686) is an antagonist of tachykinin receptor 1 (TACR1, also called neurokinin-1 (NK1R)). It is currently being investigated for treating symptoms of nausea and vomiting in motion sickness and gastroparesis, pruritis, and social anxiety disorders [102,103].

Mechanism of action: Significant levels of the neurotransmitter substance P have been found in the brainstem regions involved in vomiting, such as the nucleus tractus solitarius and the area postrema. Additionally, it has been observed in the axons and varicosities that make synaptic contact with the neurons in these regions. Substance P immunoreactivity is present in the vagal afferents of the nodose ganglion and abdominal vagus, the majority of which supply the abdominal viscera through the vagus unmyelinated afferent fibers. These results imply that substance P neurotransmission may represent a promising target for the creation of antiemetics [104]. Tradipitants inhibit the binding of substance P to NK1R in the central nervous system (CNS) by competitively binding to the receptor. In short, tradipitant’s mechanism of action is the inhibition of substance P from binding to NK1R [103]. 

Efficacy and safety: Tradipitant is being investigated as a treatment for nausea and vomiting symptoms in diabetic gastroparesis patients. From November 2016 through December 2018, a double-blind trial of 152 adults with gastroparesis studied the effectiveness of oral tradipitant 85mg given twice daily for four weeks in comparison to placebo on symptoms, with the primary outcome being the change in average nausea severity measured by the Gastroparesis Core Symptom Daily Diary (GCSI-DD) in the intent-to-treat analysis. The study concluded that the use of tradipitant resulted in significant improvement of nausea in gastroparesis patients [105]. A multicenter, randomized, double-blind, placebo-controlled phase 3 study by Vanda Pharmaceuticals started in 2019 uses the GCSI-DD as a measure of DG severity to assess the efficacy of tradipitant in treating symptoms associated with diabetic gastroparesis. This study found that participants who were given tradipitant experienced significant relief from DG-associated nausea. Patients who were given tradipitant experienced a significant reduction in their nausea score, with a decrease of 1.2, by the fourth week of treatment. In comparison, those who received a placebo only saw a decrease of 0.7 in their nausea score (P = .0099). Furthermore, the group receiving tradipitant had a significant increase in the number of days without experiencing nausea, with a 28.8% improvement, whereas the placebo group only saw a 15.0% increase (P = .0160). The greater improvement in patient symptoms under the administration of tradipitant compared to the placebo group led researchers to conclude that the use of tradipitant in the treatment of DG significantly improves the QoL in patients suffering from DG [106]. 

Clinical uses: Tradipitant is currently being investigated for its potential clinical uses in various conditions such as nausea and vomiting associated with chemotherapy, alcohol use disorder, and pruritus associated with chronic kidney disease [107]. Tradipitant’s inhibition of substance P from binding to NK1R is the mechanism of action in all its potential uses. 

Adverse effects: The common adverse effects (AEs) of tradipitant include headache, dizziness, diarrhea, and upper respiratory tract infection. Serious AEs such as liver toxicity and allergic reactions have also been reported.

Relamorelin

Drug class: Relamorelin, or RM-131, is a synthetic pentapeptide with better stability and prolonged plasma circulating half-life than native ghrelin. It exhibits potent gastric and colonic prokinetic effects by binding to the growth hormone secretagogue (GHS)-1a (or ghrelin) receptor [108,109]. 

Mechanism of action: In vivo animal studies have demonstrated a dose-dependent effect of ghrelin on gastric emptying and intestinal transit [110]. These studies have shown that ghrelin increases gastric motor function via multiple pathways including direct action on the enteric nervous system by vagal signaling, and by crossing the blood-brain barrier and affecting the vagal function in the central nervous system [111]. Relamorelin acts via binding to the growth hormone secretagogue (GHS)-1a (or ghrelin) receptor and has been shown to be nearly six times more potent than ghrelin in activating the GHS-1a receptor [108]. 

**Figure 3:**
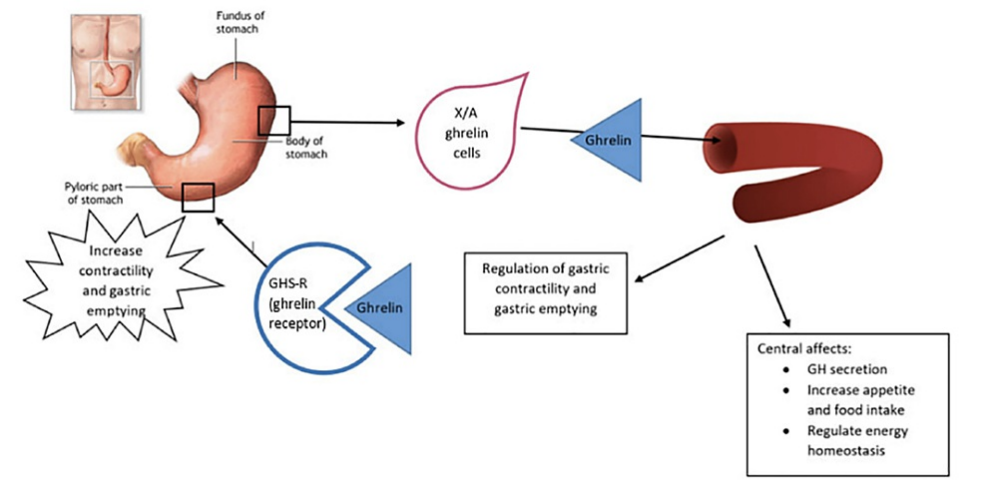
Role of Ghrelin in gastric motility. Reproduced with permission from Petri et al [112]. Copyright 2021 Elsevier.

Figure 3 attached below shows the effect of ghrelin on gastric emptying.

Efficacy and safety: There have been four randomized control trials (RCT) between 2013-2017 testing the efficacy and safety of relamorelin. Shin et al. conducted two RCTs in 2013. Both were double-blind cross-over studies where patients were given a single dose of relamorelin or placebo, but one included type 1 diabetics with documented delayed gastric emptying, and the second included type 2 diabetics with documented delayed gastric emptying. The first study showed that relamorelin significantly reduced the GCSI-DD and nausea, vomiting, fullness, and pain (NVFP) scores compared to placebo. No serious or significant AEs were seen, and hunger was the one AE with borderline significance [113]. The second study showed a significant acceleration in gastric emptying time for solids in the relamorelin group compared to the placebo and the drug was well tolerated with no serious AEs [114].

Lembo et al. conducted a multicenter, double-blinded, placebo-controlled RCT of 204 diabetic patients with gastroparesis where the patients were given twice daily dosing of relamorelin or placebo for 28 days. Compared to the placebo group, the twice-daily relamorelin group showed significantly reduced gastric emptying time as well as reduced frequency and severity of emesis. There were no serious AEs; the most common were headaches and worsening of pre-existing DM [115]. The latest RCT was conducted by Camilleri et al. in 2017 where they performed a multicenter, double-blinded, placebo-controlled RCT of 393 diabetic patients with gastroparesis who were randomly assigned into a placebo group or one of three treatment groups (receiving twice daily relamorelin at 10 ug, 30 ug or 100 ug). Longitudinal analysis showed a significant reduction in all four symptoms of diabetic gastroparesis such as nausea, postprandial fullness, abdominal pain, and bloating in all three dose groups compared to the placebo group. Reduction in the frequency of vomiting was not significant but there was a 75% reduction in drug groups and an unusual 70% reduction in the placebo group. Relamorelin significantly improved gastric emitting than baseline compared to placebo in all three dose groups [109]. 

Clinical uses: Being a potent prokinetic, relamorelin is being investigated for use in cases of gastroparesis, post-operative ileus (POI), and chronic constipation [108]. 

Adverse effects: Analysis of phase 2a and 2b trials showed that relamorelin in the 10-100 ug dose range is well tolerated. The most common AEs were headache, worsening hyperglycemia, and diarrhea. There was a significant increase in HbA1c levels and fasting blood glucose levels between baseline and the 12th week of treatment. This could be explained by potential mechanisms like inhibiting insulin release or increasing insulin resistance by increasing growth hormone release [116]. Furthermore, prokinetics like cisapride have been shown to increase early postprandial glucose [117].

Lifestyle modifications and future recommendations

Maintaining oral nutrition is a goal of treatment for mild disease. Enteral or parental nutrition will be required in the case of serious gastroparesis. Dietary advice is based on measures for optimizing the emptying of the stomach such as incorporating a diet consisting of small meals as the stomach can only absorb 1-2kcal per minute. Foods rich in fats and fibers delay gastric emptying so it is recommended to limit the intake of such meals. Blended solids or nutrient-rich liquids may empty normally, given that gastric emptying of liquids is often preserved during gastroparesis [118,119]. Therefore, it is appropriate for patients with gastrointestinal conditions to eat small, low-fat, low-fiber meals four to five times a day. By multiplying 25 kcal by their body mass in kilograms, the patient's caloric requirement can be calculated [118,119]. Smoking and alcohol should be avoided due to their potential to alter gastric emptying [120]. 

In some patients, carbonated beverages with the release of carbon dioxide may cause gastric distension; their intake should be reduced [118,119]. Patients should continue to take fluids during their meals and sit or stand for up to two hours after eating. In contrast to a normal diabetic diet, low particle dietary intake may also help with symptoms and tolerance [121].

Diabetes gastroparesis is linked to more complications, such as more hospital stays and trips to the emergency room. Between 1995 and 2004, the number of hospital admissions attributed to gastroparesis increased by 138% [60]. 

An integral part of the treatment plan is patient and family education and improved awareness of this condition. The patient's QoL, as well as their personal and social lives, are significantly impacted by the incapacitating chronic symptoms of gastroparesis. The patient should be helped to cope with his or her disability through empathy for the patient's needs, a humanistic approach from the clinical team, and psychological counseling. Patients should be informed that, in order to find the optimal treatment regimen, several medicines may be tried, and that the aim of treatment is to control rather than cure the disease. Physical conditioning, weight, and nutrition issues must be addressed in the treatment of DGP [120,122]. 

In order to have an impact on overall diabetes outcomes, it is necessary to optimize glycemic control to reduce the symptoms of DGP and improve the emptying of the stomach. Hyperglycaemia, which is likely to be mediated by decreased phasic antral contractions and induction of pyloric pressure waves, delays the emptying of the stomach even without neuropathy or myopathy. Hyperglycaemia may slow the effects of prokinetic agents. To prevent impeding stomach myoelectric regulation and motility, glucose levels should be kept below 180 mg/dL. Patient interventional strategies need to be developed for the reduction of postprandial hyperglycemia [73,123-125]. In order to implement a strategy of individual patient care, a multidisciplinary approach with a team consisting of a certified diabetes educator, a registered dietician with experience in nutritional assessment of gastroparesis and a behavioral psychologist is essential. In addition, effective partners in the patient care team should be compassionate family members and caregivers who understand the dynamics and complexity of blood glucose management in patients with gut autonomic dysfunction [125]. The development of DGP is suspected to be influenced by Inflixivic Botulinum Injection Pylorospasm. In several open-label trials botulinum toxin, a potent inhibitor of neuromuscular transmission, has been shown to improve emptying and symptoms in DGP and idiopathic gastroparesis for some months [126]. It is beneficial to eliminate medicines that exacerbate DGP [127]. 

## Conclusions

Diabetic gastroparesis was once considered to be a debilitating complication of diabetes mellitus. Advancements in research and new treatment modalities have provided a breakthrough in the treatment of DG. In this review article, we have summarized the results of various experiments and research that were conducted to test the two emerging drugs for DG, namely relamorelin and tradipidant. While a reasonable amount of data has been collected to support the use of relamorelin and tradipitant in the treatment of DG, further experiments and trials are still being conducted to assess the long-term effects of these drugs. To conclude, we can say that with the correct treatment regimens and appropriate lifestyle modifications, it is only a matter of time before DG is considered a particularly manageable sequela of DM.
